# Individualized pharmaceutical care for a patient after pancreaticoduodenectomy with trypsin replacement nutritional therapy: A case report

**DOI:** 10.1097/MD.0000000000030209

**Published:** 2022-08-26

**Authors:** Song Zhang, Qin Tan, Hanjun He

**Affiliations:** a The First People’s Hospital of Guangyuan, Guangyuan, China; b The People’s Hospital of Nanchuan, Chongqing, China.

**Keywords:** individualized medication, pancreatic enzyme replacement therapy, pancreaticoduodenectomy, pancreatin enteric-coated capsules, pharmaceutical care

## Abstract

Optimal nutritional therapy for pancreaticoduodenectomy (PD) has been debated; however, little is known about key points of pancreatin enteric-coated capsule administration, a critical component of the PD treatment regimen. Patients often report elevations in tablet platoon and steatorrhea, and steatorrhea may adversely affect nutritional therapy for PD. Herein, we report a case of individualized pharmaceutical care for a patient after PD with trypsin replacement nutritional therapy.

**Patient concerns and diagnosis:**

After PD with trypsin replacement nutritional therapy, the patient developed acute steatorrhea.

**Intervention:**

Individualized pharmaceutical care was provided by clinical pharmacists to address intolerance to pancreatin enteric-coated capsules following PD.

**Outcomes:**

The clinical pharmacist’s integration into the patient’s treatment plan enhanced pharmacotherapy optimization, especially through pharmacokinetic monitoring and interventions related to nutritional therapy.

**Lesson:**

Pharmaceutical care by clinical pharmacists aids in ensuring the safety and efficacy of drugs and nutritional treatment. Clinical pharmacists should be members of the nutrition support team.

## 1. Introduction

Nutrition and weight are important outcomes linked to reduced morbidity, improved condition, and better survival in patients with pancreaticoduodenectomy (PD). For patients with PD who are pancreatic insufficient, taking pancreatic enzyme supplements is an important component of their treatment regimen. However, digestive tract remodeling is common in PD, resulting in decreased digestive function and loss of pancreatic enzyme secretion. The complex regulatory mechanism among gastric, duodenum, and pancreatic secretion in the digestive chain is destroyed and the gastric contents cannot be neutralized to the optimal pH level, resulting in decreased intestinal PH. In such cases, patients require long-term pancreatic enzyme replacement therapy due to a lack of duodenal intestinal secretion function resulting from the changes in intestinal pH level and accelerated digestive tract peristalsis.

Pancreaticoduodenectomy is primarily treated with enteric-soluble capsules, and a few cases have been successfully treated. However, PD cases of pancreatin deficiency, vitamin and mineral deficiency, steatorrhea, weight loss, and nutrient deficiency have been reported.^[1]^ Therefore, it is important to pay close attention to the nutritional status of patients with PD and correct malnutrition reasonably. Patients often report elevations in tablet platoon and steatorrhea, and steatorrhea may adversely affect nutritional therapy for PD. This suggests that individualized treatment plans for pancreatin replacement therapy are needed. Additionally, the early recognition and differential treatment of pancreatic ferment in patients with malnutrition are necessary. Individual treatment with replete trypsin enteric-soluble capsule supplementation and nutrition support therapy can reduce the risk of a PD and improve the prognosis.

In this report, we detailed the individualized pharmaceutical care of a patient with PD provided by clinical pharmacists to promote personalized nutrition therapy after PD. We analyzed the dosage form characteristics of pancreatic enzyme enteric-soluble capsules, and implemented appropriate personalized medication guidance for the patient based on the drug characteristics.

## 2. Case presentation

A 20-year-old man presented to the general surgery department after a car accident. Due to the car accident, he underwent surgical treatment of the common hepatic artery, splenic artery, bilateral renal artery, superior mesenteric artery angiography + gastroduodenal artery embolization in a local hospital, and laparotomy, subtotal gastrectomy, total PD, cholecystectomy, splenectomy, and bilioenterostomy. The postoperative diagnoses were as follows: pancreatic rupture, duodenal damage, severe traumatic pancreatitis, colonic trauma, contusion of intestine, retroperitoneal hematoma, acute diffuse peritonitis, septic shock, pulmonary infection, acute liver injury, spinal cord injury, and hypoproteinemia. In addition, the patient had a history of smoking and alcoholism.

At the time of admission, his height was 174 cm, weight was 55.0 kg, and body mass index was 18.2 kg/m^2^. His weight had fallen by 15 kg within approximately 1 month. He scored 5 points on the Nutritional Risk Screening tool (NRS2002), which is used for the nutritional assessment of hospitalized patients with a respiratory disorder to guide the rational use of nutritional support. He was graded on the following 3 signs in the operational criteria proposed by the NRS2002 for the assessment of nutritional risk: age (20 years, grade 0); fast weight loss (his weight dropped from 60 to 55 kg within 1 month, grade 3); and disease severity (moderate, grade 2). Accordingly, the patient was diagnosed with malnutrition and required nutritional treatment.

## 3. Treatment and clinical pharmacist monitoring

### 3.1. Treatment

The principal medical treatments included symptomatic treatment, anti-infection, nutritional therapy, and surgery. At 27 days, he scored 5 points on the NRS2002. Thus, a nutritional risk still existed and the patient required continued nutritional therapy. A gradual transition from parenteral nutrition (PN) to enteral nutrition (EN) was recommended to restore his intestinal function gradually. His swallowing function gradually recovered, the abdominal drainage was reduced, and EN was started. Initially, he was given partial PN and partial EN for 6 days. As partial PN, 250 mL of compound amino acids (18AA-II) was administered once daily. As partial EN, a nutrition solution with intact protein-based EN powder at a concentration of 20% to 25% was prepared and pumped into a nasogastric tube at a constant speed. Subsequently, according to the patient’s gastrointestinal tolerance, he was gradually transitioned to total EN, that is, the compound amino acid injection (18AA-II) was replaced by whey protein powder. At this time, bilateral lower limb weakness and reduced activity were noted. The muscle strength of the right limbs was grade II and the muscle tone was reduced.

At 40 days, he scored 5 points on the NRS2002. Thus, a nutritional risk still existed and the patient required continued nutritional therapy. The muscle strength of the right limbs was grade II, his vital signs were stable, and a cardiopulmonary physical examination showed no obvious abnormalities. He had an abdominal depression, with incision length of approximately 13 cm, width of approximately 2.5 cm, and depth of approximately 2.0 cm. There was no obvious pus in the incision and the wound surface was rosy, with rich granulation tissue. The patient received EN. At this time, the patient asked for a light diet under consultation.

In consideration of his PD, he was given pancreatic enzyme enteric-soluble capsules with his meals. We adjusted the patient’s EN regimen to a light diet with supplementary EN powder and pancreatic enzyme enteric-soluble capsules (dose adjusted with diet) for 2 days. The patient developed diarrhea with obvious fecal odor, and the whole drug capsule could be seen in the stool. Therefore, fatty diarrhea was suspected, but the occurrence of a new lesion could not be ruled out. However, the patient did not have fever, chills, dizziness, headache, unconsciousness disorders, skin petechiae, petechiae, bleeding tendency, abdominal pain, abdominal distension, vomiting, or other symptoms. According to the patient’s condition and physiological changes, the administration plan for pancreatic enzyme enteric-soluble capsules was adjusted in combination with the characteristics of the dosage form of pancreatic enzyme enteric-soluble capsule.

Interestingly, after individualized pancreatic enzyme enteric-soluble capsule and nutritional treatment, his vital signs and laloplegia significantly improved within 2 days. His gastrointestinal function continued to improve and his physical activity gradually recovered within 2 weeks. Subsequently, the patient, whose condition was relatively stable, was given individualized pancreatic enzyme enteric-soluble capsules orally 3 times daily, and he was transitioned from EN to a food homogenate (low-salt and low-fat diabetes homogenate).^[2]^ The patient had no fever, abdominal pain, abdominal distension, vomiting, or other symptoms. His urine was normal and stool was unformed, with 3 to 5 bowel movements per day. He showed improved nutritional status (total protein, 70.2 g/L; albumin, 36.3 g/L), with no significant change in body weight. His condition was stable and he continued to receive other treatments. As expected, his nutrition-related laboratory indicators and electrolytes gradually returned to normal levels. Laboratory data are shown in Table 1.

**Table 1:** Nutritional treatment program.

Treatment stage	Time	The clinical situation	Nutritional regimen	Total calories and protein
Stage 1 treatment	D6–D9	After 3–4 d in the ICU, the patient’s condition was improved on day 6 and he was transferred to the general ward for intensive care. At this time, the patient was conscious, without fever, chills, or other discomfort. Physical examination revealed the following: heart rate, 102 beats/min; blood pressure, 125/58 mm Hg; 1 chest catheter could be seen on the right chest wall; and the drainage tube was unblocked. In the continuous negative pressure drainage of the abdominal incision, the drainage was unobstructed, 4 drainage tubes and 1 fistula tube were seen in abdominal cavity, abdominal drainage tubes were unobstructed, abdominal muscle was tense, fecal material flowed out of abdominal wall incision, and bowel sounds were not heard. The right hip and lower limb were swollen. The right lower limb was fixed with a special brace. His muscle strength was approximately grade 2 and NRS2002 score was 5. He received PN.	Fat emulsion, amino acids (17), glucose (11) injection (1440 mL)	Total calories, 1000 kcal; protein, 34 g; non-protein calories, 900 kcal
	D9–D14	On day 9, the patient’s heart rate was increased (approximately 120 beats/min), abdomen was flat, no gastrointestinal type or peristaltic wave was observed, the abdominal incision continued to attract negative pressure, the skin in the upper part of the incision was confluent, intestinal fluid outflow could be seen in the middle and lower parts of the incision, his whole abdomen was slightly tender, without obvious rebound pain or tension, and his liver function results were as follows: total protein, 56 g/L; albumin, 32.5 g/L. On day 12, the patient indicated that his abdominal pain was significantly better and cough and sputum were relieved. There was no abdominal distention, vomiting, or other discomfort. He had mental distress, poor sleep, and unformed stool. The abdominal incision still had fecal-like material outflow and his whole abdomen was slightly tender, without obvious rebound pain and tension. His right lower limb continued to be fixed with nail shoes. The muscle strength of the right lower limb was grade 3, and the blood flow of the extremity was reasonable. Re-nutrition assessment with NRS2002 resulted in a score of 5, the patient’s condition was stable, and PN treatment was continued.	Fat emulsion, amino acids (17), glucose (11) injection (1920 mL)	Total calories, 1400 kcal; protein, 45 g
	D14–D27	On day 14, the patient had slight pain around the abdominal incision. There was no abdominal distention, vomiting, or other discomfort. His spirit and sleep were good, urine was normal, and stool was unformed. His liver function results were as follows: total protein, 70.2 g/L; albumin, 36.3 g/L. He was still in the state of malnutrition and the gastrointestinal tract was not recovered. PN treatment was continued, and PN protein was increased to 87.5 g.	Fat emulsion, amino acids (17), glucose (11) injection (1920 mL) + compound amino acids (18AA-II, 8.5% 500 mL), 42.5 g	Total calories, 1570 kcal; protein, 87.5 g
Stage 2 treatment	D27–D40	On day 27, the patient complained of numbness in the right lower limb, occasional pain in the abdomen that was tolerable. There was no abdominal distention, vomiting, palpitations, chest tightness, or other discomfort. His mental state, sleep, and urine were as usual, and he had unformed stool. His abdomen had an incision approximately 17 cm in length, with a large amount of fresh granulation tissue. There was still a small amount of fecal material flow in the mass samples. The incision under the xiphoid process was largely restored. There was slight skin redness around the abdominal incision, without obvious rebound tenderness. Tension, liver, and spleen function were unchanged. The right lower limb was fixed in nail shoes. His muscle strength was grade II, without obvious abnormities. His endotrophic tolerance assessment score was 1 and acute gastrointestinal injury was grade II. He continued progressive ONS enteral therapy.	Fat emulsion, amino acids (17), glucose (11) injection (1440 mL) + enteral nutritional powder (SP) 50 mL/d, with added amount to 750 mL	PN total calories, 1000 kcal; protein, 34 g; EN (SP), 1 kcal/mL; protein, 0.04 g/mL
Stage 3 treatment	D40–D42	On day 40, the patient complained of pain in the right lower limb. There was no abdominal discomfort after eating, cough, sputum, or obvious abdominal distention. He had regular urination and unformed stool. On day 42, the patient had diarrhea, with obvious fecal odor. The whole drug capsule could be seen in the feces; thus, fatty diarrhea was considered. In confirmation of the diagnosis, the patient did not have fever, chills, dizziness, headache, unconsciousness disorders, skin petechiae, petechiae, bleeding tendency, abdominal pain, abdominal distension, vomiting, or other symptoms.	Continued ONS sequential therapy, trypsin replacement therapy, gradually transitioning to whole protein type EN powder	Heat target: 20–25 kcal/kg; protein, 1.5 g/kg
	D42–D45	On day 42, his NRS2002 score was 5. The patient still required continued nutritional therapy. The patient complained of pain in the right lower limb. There was no abdominal discomfort after eating, cough, sputum, or obvious abdominal distention. He showed regular urination, fatty diarrhea, and a little fecal material outflow in the drainage. On day 45, the patient had no fever, abdominal pain, abdominal distension, vomiting, or other symptoms. His urine was normal and stool was unformed, with 3–5 bowel movements per day. He showed improved nutritional status (total protein, 70.2g/L; albumin, 36.3 g/L), no significant change in body weight, sand table condition. He continued to receive other treatment.	Bland diet, supplementary EN powder, trypsin enteric-soluble capsules (individualized treatment plan)	Heat target: 20–25 kcal/kg; protein, 1.5 g/kg

EN = enteral nutrition, ICU = intensive care unit, NRS2002 = Nutrition Risk Screening, ONS = oral nutritional supplement, PN = parenteral nutrition.

At 50 days, the patient was transferred to the Department of Rehabilitation Medicine for further rehabilitation training, and his limb muscle strength increased gradually.

### 3.2. Pharmaceutical care by clinical pharmacists

Pharmaceutical care by clinical pharmacists was followed to ensure the safety and efficacy of the patient under drug and nutritional treatment.^[3]^ The pharmaceutical care by clinical pharmacists is detailed in Table 2.

**Table 2:** Clinical pharmaceutical care.

Time	Suggestions and guardianship content	Recommendation reasons	Result
D6	Start nutritional therapy and strictly monitored the patient’s changes in vital signs and laboratory indicators	The hemodynamics of the patient was stable after the trauma, a nutritional risk existed, and patient required nutritional therapy.	Physicians took advice
D9	Increase nutritional healing target amount	After trauma, the patient was in a stable period and his nutritional demand was increasing. The current nutritional plan did not meet the target requirement.	Physicians took advice
D14	After nutritional assessment, administer 8.5% 500 mL of compound amino acids (18AA-II) once daily as partial parenteral nutrition	The patient was in the recovery stage of trauma, with negative protein balance for a long time. He required a 1.5-g/kg protein supplement.	Physicians took advice
D27	Tentatively initiate ONS, with short peptide enteral nutrition preparations	The patient’s gastrointestinal function had gradually recovered. In order to speed up the patient’s recovery, his intestinal mucosa need to be protected and bacterial community transfer needed to be prevented.^[2]^	Physicians took advice
D40	Continue ONS sequential therapy, trypsin preparation replacement therapy, and gradually transition to a treatment regimen of whole protein enteral nutrition powder, with trypsin preparations	After ONS, the gastrointestinal function of the patient had gradually recovered. Considering the economy and living ability of the patient, the ONS formula was changed and complementary food was gradually added.	Physicians took advice
D42	Administer trypsin enteric-soluble capsules (individualized treatment plan)	For patients with radiography and fatty diarrhea, individualized medication is important.	Physicians took advice

ONS = oral nutritional supplement.

Regarding the dosing regimen, clinical pharmacists designed an individualized plan for the administration of pancreatin enteric-soluble capsules, with consideration of drug instructions, relevant guidelines, references, and the patient’s status. The clinical pharmacists initially recommended 40,000 to 50,000-IU meals and 10,000 to 20,000-IU snacks, in accordance with the 2018 International Study Group on Pancreatic Surgery position paper on nutritional support and treatment for adult patients with pancreatic surgery.^[4]^ This could be increased to 72,000 to 75,000-IU meals and 36,000 to 50,000-IU supplementary meals. For adults, the recommended initial dose is 40,000-IU meals and 20,000-IU snacks, which can be increased according to the individual dose to the maximum dose of 75,000 to 80,000 IU lipase/meal. Further, it was recommended that the patient take trypsin preparations with meals.^[5]^

As per Creon^[6]^ instructions for the supplementary treatment of exocrine pancreatic insufficiency, the dosage was adjusted according to dietary fat, presence of fatty diarrhea, and other clinical symptoms. Based on the patient’s condition, postoperative fatty diarrhea could easily occur and lipase was the most needed supplement. Thus, the amount of pancreatic enzyme supplement was mainly calculated by lipase. As the patient weighed 45 kg, the total daily supplemental dose of pancreatic lipase was estimated as <450,000 IU (10,000 IU*45 kg = 450,000 IU). As each pancreatic enzyme enteric-soluble capsule contains about 10,000 IU of lipase, about 4 to 6 capsules were required for meals and 2 to 4 capsules were required for supplementary meals. In addition, as after most gastrostomies, gastrointestinal peristalsis was strengthened, and pancreatic enteric-soluble capsules could not be dissolved in the stomach; thus, the capsules were opened and mixed with acidic food (such as applesauce, fruit juice, and jam; at pH ≤ 4.5).^[7]^ To avoid damaging enteric-soluble particles, particles were 1 mm in size.

## 4. Discussion

We reported a case of individualized pharmaceutical care of a patient with PD with trypsin replacement nutritional therapy. In the present case, common hepatic artery, splenic artery, bilateral renal artery, and superior mesenteric artery angiography, and gastroduodenal artery embolization were performed at a local hospital due to a car accident. Additionally, laparotomy, subtotal gastrectomy, total PD, cholecystectomy, splenectomy, biliojejunostomy, and gastroenterojejunostomy were performed.^[5]^ Thus, in the present case, the patient’s gastric resection, full pancreaticoduodenal resection, gallbladder resection, and spleen, gallbladder, intestinal anastomoses in the postoperative reconstruction of the digestive tract caused poor digestive function, which further led to a lack of pancreatic enzyme secretion, decreased intestinal PH, and poor appetite. These factors ultimately resulted in malnutrition and digestive enzyme deficiency. Due to the lack of duodenal and intestinal secretion function, the patient required long-term pancreatic enteric-soluble capsule replacement therapy.^[5]^

After routine pancreatin enteric-soluble capsule supplement therapy, the patient presented with such phenomena as tablet platoon and steatorrhea, raising several questions. Are there suitable disintegrating conditions for trypsin enteric-soluble capsules? Is there sufficient disintegration time? Is trypsin activity affected? Clinical pharmacists provided individualized medication guidance and pharmaceutical care to the patient to address these issues.

### 4.1. The characteristics of enteric capsule dosage forms

Enteric capsules^[8]^ are characterized by delayed-release formulations with enteric coating and are insoluble in gastric juice. They have some risk, but play an important local or systemic therapeutic role. Enteric coating materials include hexadecyl alcohol, dimethyl silicone oil, hydroxypropyl methylcellulose, phthalate acetate, polyethylene glycol, and triethyl citrate.^[9]^ The principal advantages of the use of enteric capsules are as follows: avoidance of strong gastric mucosa stimulation caused by drugs and resulting in nausea, vomiting, and other adverse reactions; avoidance of gastric acid damage to enzymes and sensitive drugs; and high bioavailability, as drugs mainly absorbed by the small intestine and colon are transferred at the highest concentration possible for local or systemic therapy. Enteric-soluble capsules/microparticles protect pancreatic enzymes from irreversible inactivation at the low pH of gastric acid and inadequate release due to pathophysiological changes in the patient.

Because of the present patient’s digestive tract environment (pH value in the digestive tract, digestive fluid composition, digestive peristalsis function and speed, etc), tablet placement was not affected by the quality of the drug itself. In addition, the patient’s small intestine was partially resected and intestinal peristalsis was fast. Accordingly, it was difficult for the internal drug components to be released completely and exert their effects; thus, a vicious circle formed, further diminishing the role that the drug components were intended to play. Therefore, the postoperative changes in intestinal pH and gastrointestinal peristalsis were accelerated. In such cases, enteric-soluble capsules cannot have a curative effect, and patients with platoon tablets are prone to pancreatic enzyme deficiency, vitamin and mineral deficiency, steatorrhea, weight loss, and nutrient deficiency.

### 4.2. Trypsin activity

The existing trypsin did not finish bioequivalence studies.^[10]^ Most enteric-soluble designs (capsules or microparticles) to protect the trypsin against the gastric acid at pH < 4 and release activity in intestine canal at pH levels ranging 5 to 5.5. Enteric-coated particles mix with food and work better than enteric-coated tablets. The close association between the mean retention time and pH within duodenum organs is essential to the activity function of trypsin. Duodenal pH is 1 of the important factors determining the release of trypsin by enterolysin particles. Enterolysin particles can only be released further in the intestine after PD, affecting absorption. In addition, the infant gastrointestinal tract is an imperfect system, but will also affect absorption.

Related studies have found that the pH of the distal duodenum after eating is 5.75, which is lower in patients with pancreatic secretion disorder.^[8,11]^ A study by Gan et al reported the dissolution time of 5 enteric-coated particles/tablets at pH = 4 to 6. The dissolution rates of Creon, Creon Forte, Pancrease HL, and Panzytrat in USP buffer at 128 r/min at pH 4 to 6 and 37ºC were measured at 280 nm. The dissolution rate of Panzytrat and Pancrease HL was 43% within 30 minutes (pH = 5.0), 50% within 30 min (pH = 5.2), and 90% within 30 minutes (pH = 5.6). The solubility was 90% at pH ≥ 5.8 for all preparations. There was no optimal dissolution curve for the treatment of pancreatic exocrine insufficiency. However, the novel preparation, Pancrease HL, with high lipase content was released at pH ≤ 5.4, with a slightly better dissolution curve than the other preparations. Additionally, mono-fatty glycerides and fatty acids were absorbed in the distal duodenum and proximal jejunum with Creon 25000; at pH = 5.2 and 120 minutes, the release was 14% and at pH = 5.4 to 5.6 and 30 minutes, the release was 85%.^[8]^ Creon 25000 has high sensitivity to the pH level and fast release, but it is also necessary to consider gastric acid damage to enzymes, especially when the stomach pH level can be rapidly reduced by 4 or less within 30 minutes after eating, and lipase is released simultaneously, with reduced activity reduced at pH = 4.5. Creon 25000 is more suitable for patients after gastrectomy. Treatment with a proton pump inhibitor or H2 antagonist can increase the pH level of the small intestine.

## 5. Summary

In summary, patients with PD often have complications of malnutrition, which may result in nutrient deficiency-related pancreatic enzyme disorders, such as fatty diarrhea. Therefore, it is important to pay close attention to the nutritional status of patients with PD and correct malnutrition reasonably. There were delays in the diagnosis and treatment of fatty diarrhea in the present case, suggesting that the importance of individual treatment for pancreatin replacement therapy. Additionally, the early recognition and differential treatment of pancreatic ferment in patients with malnutrition are necessary, and individual treatment with replete trypsin enteric-soluble capsule supplementation and nutrition support therapy can reduce the risk of a PD and improve the prognosis.

The key points that should be considered during treatment are as follows: physicians should accurately assess the nutritional risk of patients, and determine the nutritional plan and timing of treatment; reasonable nutrition and pancreatic enzyme supplementation should be selected according to the changes in the patient’s condition after the recovery of gastrointestinal function; and individualized drug treatment according to the characteristics of trypsin preparations and the patient’s condition should be guided by clinical pharmacists. In addition, clinical pharmacists should participate in the treatment plan throughout the whole process, develop individualized medication for patients, and conduct medication education for patients, which is conducive to medication compliance, resulting in sustainable benefits to clinical treatment.

## Acknowledgments

We would like to acknowledge support by The First People’s Hospital of Guangyuan. We would also like to thank Editage (www.editage.com) for English language editing. All aforementioned entities have given permission to be named in the published manuscript.

## Author contributions

**Conceptualization:** Song Zhang.

**Data curation:** Song Zhang, Qin Tan.

**Supervision:** Hanjun He.

**Writing – original draft:** Song Zhang, Qin Tan.

**Writing – review & editing:** Hanjun He.

**Data curation:** Song Zhang, Qin Tan, Hanjun He.

**Formal analysis:** Song Zhang, Qin Tan.

**Project administration:** Song Zhang, Qin Tan, Hanjun He.

**Writing – original draft:** Song Zhang.
